# Human-Specific SNP in Obesity Genes, Adrenergic Receptor Beta2 (*ADRB2*), Beta3 (*ADRB3*), and PPAR γ2 (*PPARG*), during Primate Evolution

**DOI:** 10.1371/journal.pone.0043461

**Published:** 2012-08-24

**Authors:** Akiko Takenaka, Shin Nakamura, Fusako Mitsunaga, Miho Inoue-Murayama, Toshifumi Udono, Bambang Suryobroto

**Affiliations:** 1 Department of Health and Nutrition, Faculty of Health and Human Life, Nagoya Bunri University, Inazawa, Aichi, Japan; 2 Department of Cellular and Molecular Biology, Primate Research Institute, Kyoto University, Inuyama, Aichi, Japan; 3 Wildlife Research Center of Kyoto University, Kyoto, Kyoto, Japan; 4 Kumamoto Sanctuary, Kyoto University, Uki, Kumamoto, Japan; 5 Department of Biology, Faculty of Mathematics and Natural Science, Bogor Agricultural University, Bogor, Indonesia; University of Florence, Italy

## Abstract

Adrenergic-receptor beta2 (*ADRB2*) and beta3 (*ADRB3*) are obesity genes that play a key role in the regulation of energy balance by increasing lipolysis and thermogenesis. The *Glu27* allele in *ADRB2* and the *Arg64* allele in *ADRB3* are associated with abdominal obesity and early onset of non-insulin-dependent diabetes mellitus (NIDDM) in many ethnic groups. Peroxisome proliferator-activated receptor γ (*PPARG*) is required for adipocyte differentiation. *Pro12Ala* mutation decreases PPARG activity and resistance to NIDDM. In humans, energy-expense alleles, *Gln27* in *ADRB2* and *Trp64* in *ADRB3*, are at higher frequencies than *Glu27* and *Arg64*, respectively, but *Ala12* in *PPARG* is at lower frequency than *Pro12*. Adaptation of humans for lipolysis, thermogenesis, and reduction of fat accumulation could be considered by examining which alleles in these genes are dominant in non-human primates (NHP). All NHP (*P. troglodytes*, *G. gorilla*, *P. pygmaeus*, *H. agilis* and macaques) had energy-thrifty alleles, *Gly16* and *Glu27* in *ADRB2*, and *Arg64* in *ADRB3*, but did not have energy-expense alleles, *Arg16*, *Gln27* and *Trp64* alleles. In *PPARG* gene, all NHP had large adipocyte accumulating type, the *Pro12* allele.

**Conclusions:**

These results indicate that a tendency to produce much more heat through the energy-expense alleles developed only in humans, who left tropical rainforests for savanna and developed new features in their heat-regulation systems, such as reduction of body hair and increased evaporation of water, and might have helped the protection of entrails from cold at night, especially in glacial periods.

## Introduction

The polymorphisms of adrenergic receptor genes have been focused on because these receptors have important roles in lipolysis and thermogenesis and cause differences in energy expenditure. Adrenergic-receptor beta3 (ADRB3) is located mainly on the surface of visceral and brown adipose cells and promotes lipolysis and thermogenesis by noradrenaline release from the sympathetic nerves stimulated by cold temperature or food consumption [1], [2]. *Trp64Arg* mutation of ADRB3 is associated with lower resting metabolic rate [3], abdominal obesity [4], [5], weight gain [6], and difficulty losing weight [7]. Adipose cells with ADRB3 of *Trp64/Arg64* or *Arg64/Arg64* showed 2/3-fold reduced ability to produce intracellular cAMP [8] and lipolytic glycerol [9] compared with those with *Trp64/Trp64*. The frequency of *Trp64Arg* variant was examined in several ethnic groups [3], [10]–[17].

It is known that ADRB2 stimulates lipolysis in adipose cells as well as ADRB3 [18]. Three polymorphisms of *ADRB2*, *Arg16Gly*, *Gln27Glu*, and *Thr164Ile*, were studied in humans [19]. The *Glu27* allele has a tendency to increase BMI, body fat mass, fat cell volume, and waist∶hip ratio [20]–[23], as well as type II diabetes [24], and to suppress lipid oxidation [25]. Male African-Americans and Caucasians with *Gly16* of *ADRB2* were found to gain weight more from childhood to young adulthood than those with *Arg16* [26]. However, the contribution of *Gly16* to obesity is controversial. From the analysis of nucleotide sequences of a chimpanzee and haplotypes of *ADRB2* and *ADRB3* in humans, it was proved that *Gly16*, *Glu27*, and *Thr164* in *ADRB2* and *Arg64* in *ADRB3* are ancient types. In *ADRB2*, substitution of the energy-expense type, *Gly16∶Gln27* (GC haplotype), for *Gly16∶Glu27* (GG haplotype) occurred first 1.9 million years ago (Ma), then substitution *Arg16∶Gln27* (AC haplotype) occurred, and then there was recombination between some GG and AC haplotypes [27], [28].

Little information has been accumulated about alleles of *ADRB2* or *ADRB3* in NHP, except for the determination of the *Arg64* type in fifteen obese M. mulatta [29].

Peroxisome proliferator-activated receptor γ (PPARG) is required for adipocyte differentiation from precursor cells. PPARG forms a heterodimer with retinoid receptor, binds to peroxisome proliferation response element of the target genes in the precursor cells, and activates differentiation into small size adipocytes. These small size adipocytes secrete leptin and/or adiponectin, a factor that increases insulin sensitivity. However, upon consumption of a high-fat diet, PPARG induces adipocytes to transform from small size to large size by accumulating fat, which secrete insulin-resistance factors, such as TNFα, resistin, and free fatty acids [30], [31]. *Pro12Ala* mutation was found in *PPARG* and the *Ala12* allele frequency was found to be high in Caucasians (0.12) and low in Asians (0.01) [32]. The transcriptional activity of PPARG with *Ala12* was lower than that with *Pro12* when these were stimulated by the ligand of PPARG, thiazolidinedione [33]. *Pro12Ala* substitution was found to be associated with lower body mass index. In an obese group, subjects with *Ala12* were more insulin-sensitive than those with *Pro12* [33]. The frequency of *Ala12* allele in type 2 diabetic Japanese subjects (0.018) was significantly lower than that in a healthy group (0.043) [34].

To determine whether the appearance of energy-expense-type alleles in these genes was specific for humans, we examined SNP for the 16^th^ and 27^th^ amino acids of *ADRB2*, the 64^th^ amino acid of *ADRB3*, and the 12^th^ amino acid of *PPARG* in NHP (30 *Pan troglodytes*, 8 *Gorilla gorilla*, 17 *Pongo pygmaeus*, 15 *Hylobates agilis* and 108 macaques).

## Results

**Figure 1:**
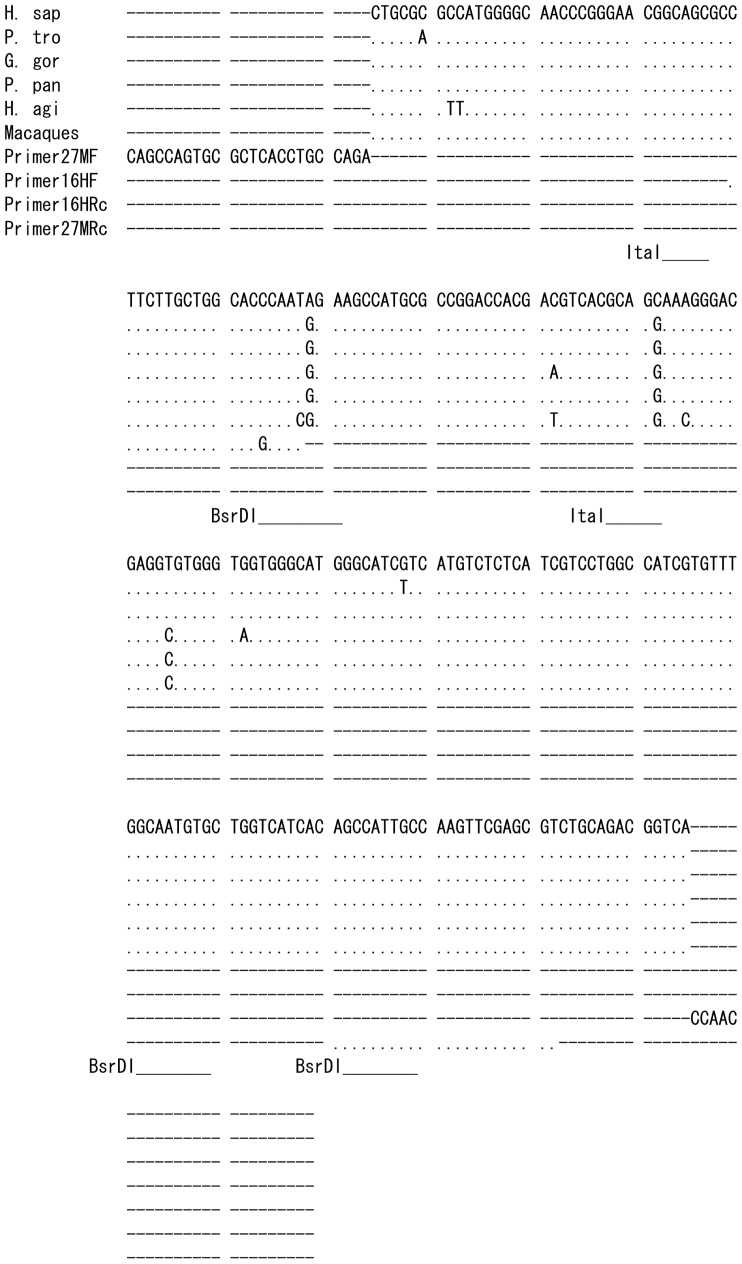
Nucleotide sequences in *ADRB2* of humans and NHPs. Primers 16HF and 16HRc (Rc means the complementary sequence of reverse primer) indicate a primer set used for the PCR to amplify the region for the 16^th^ amino acid in hominoids. One nucleotide of primer 16HF was changed to create the restriction site of *BsrD*I. Primers 27MF and 27MRc indicate a primer set used for the PCR to amplify the region for the 27^th^ amino acid in macaques. The underlines show the restriction site (GCAATGNN) with *BsrD*I for the 16^th^ amino acid and the restriction site (GCNGC) with *Ita*I for the 27^th^ amino acid. The nucleotide sequences for hominoids were determined from *G. gorilla* (DDBJ Accession No. AB669098), *P. pygmaeus* (AB669099) and *H. agilis* (AB669100), and obtained from the Ensembl database for *ADRB2* of *H. sapiens* (ENSG00000169252) and *P. troglodytes* (ENSPTRG00000017391). All macaques, *M. fascicularis*, *M. fuscata*, *M. nemestrina*, *M. radiata* (AB669101∼AB669104, respectively) and *M. mulatta* (ENSMMUG 00000002214), show the same sequence.

**Figure 2:**
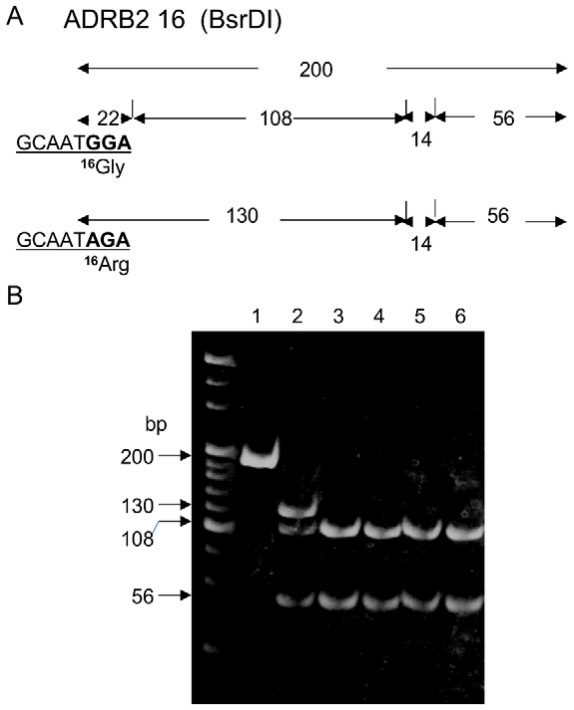
All hominoids had *Gly16* allele in *ADRB2*. A) Restriction map of *ADRB2* for the 16^th^ amino acid digested with *BsrD*I (GCAATGNN). This restriction map was predicted from the nucleotide sequences of hominoids (Fig. 1). B) RFLP patterns of PCR products of *ADRB2* for the 16^th^ amino acid digested with *BsrD*I in hominoids. Lane 1: PCR product of a human; not digested (200 bp). Lane 2: Fragments of human *Arg16/Gly16* (130,108 and 56 bp (22 and 14 bp fragments were undetectable)). Lane 3 to lane 6: Fragments from *P. troglodytes*, *G. gorilla*, *P. pygmaeus*, and *H. agilis*, respectively (108 and 22 bp instead of 130 bp).

The nucleotide sequences, the predicted restriction map, and obtained restriction fragment length polymorphism (RFLP) patterns including codon 16 of *ADRB2* are shown in Figures 1, 2A and 2B. All non-human hominoids showed a 108 bp fragment instead of a 130 bp fragment, indicating homozygosity for the *Gly16* allele, as shown in Table 1. Since the nucleotide was substituted from T to C, restriction site with *BsrD*I was not retained in the five macaques (Fig. 1), and we did not perform the RFLP method for these monkeys. However, it was confirmed by nucleotide sequence analysis that the target 16^th^ amino acid was *Gly* in each individual of five species of macaques.

**Table 1:** Frequencies of the thrifty type amino acids in *ADRB2*, *ADRB3* and *PPARG* of non-human primates.

Species	*ADRB2* 16		*ADRB2* 27		*ADRB3* 64		*PPARG* 12	
	*n*	Frequency of *Gly16*	*n*	Frequency of *Glu27*	*n*	Frequency of *Arg64*	*n*	Frequency of *Pro12*
***P. troglodytes***	30	1.0	30	1.0	30	1.0	30	1.0
***G. gorilla***	8	1.0	8	1.0	8	1.0	8	1.0
***P. pygmaeus***	11	1.0	17	1.0	17	1.0	14	1.0
***H. agilis***	15	1.0	15	1.0	15	1.0		ND
***Macaques***	5	1.0	108	1.0	108	1.0	93*	1.0
***H. sap*** ** (African)**	226	0.50^[94]^	120	0.18^[95]^	49	0.12^[3]^	53	0.97^[32]^
***H. sap*** ** (Europian)**	140	0.64^[20]^	140	0.40^[20]^	48	0.08^[3]^	26	0.88^[32]^
***H. sap*** ** (Asian)**	508	0.49^[24]^	508	0.08^[24]^	642	0.31^[3]^ **	50	0.99^[32]^

Macaques are *M. mulatta*, *M. fuscata*, *M. fascicularis*, *M. nemestrina*, and *M. radiata*.

* (*M. mulatta*, *M. fuscata* and *M. fascicularis*). ND:not detected.

** Pima Indians.

**Figure 3:**
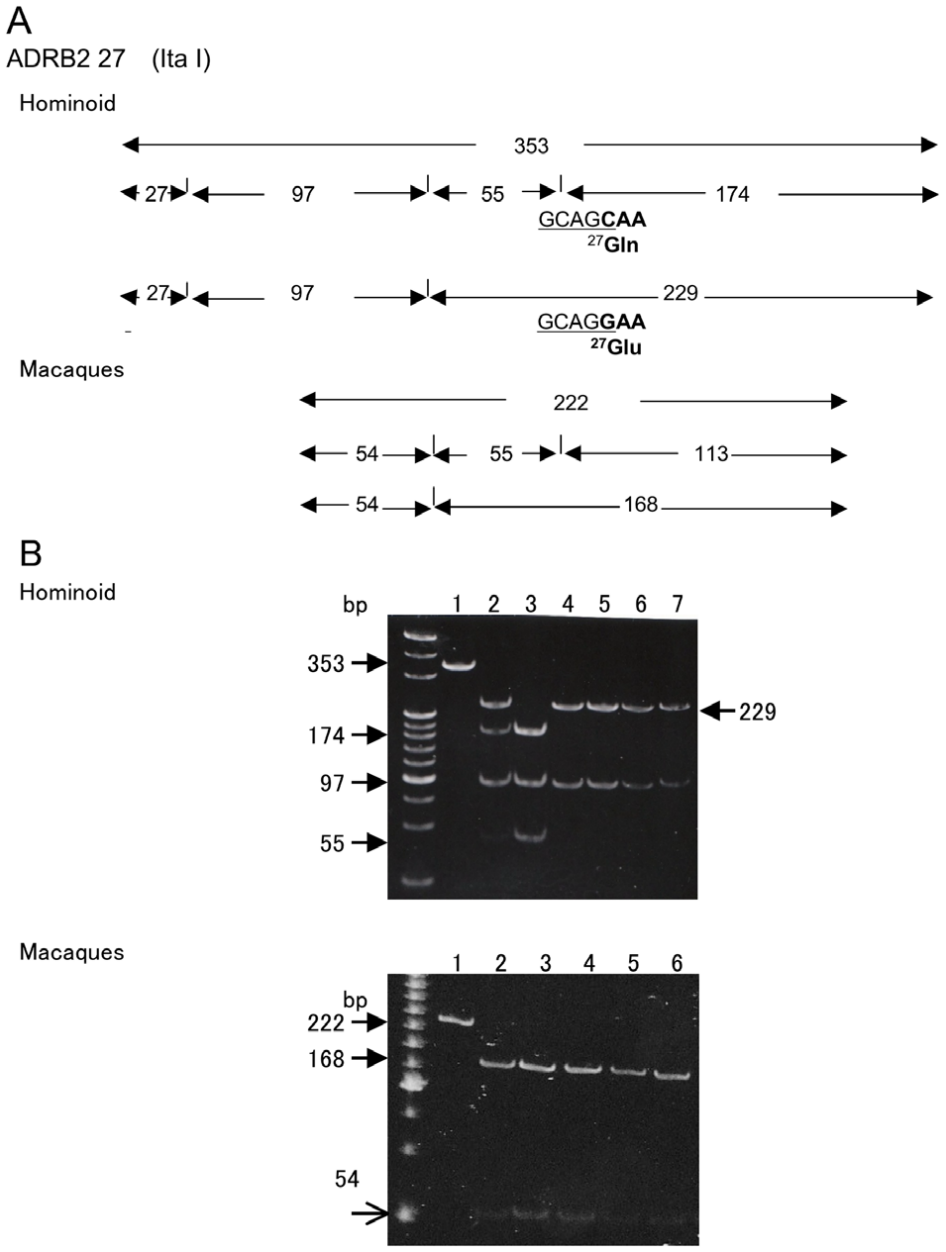
All NHP had *Glu27* allele in *ADRB2*. A) Restriction map of *ADRB2* for the 27^th^ amino acid digested with *Ita*I. This restriction map was predicted from the nucleotide sequences of humans (Ensembl database ENSG00000169252) and *P. troglodytes* (Ensembl database ENSPTRG00000017391) and from obtained macaque nucleotide sequences (Fig. 1). B) RFLP patterns of PCR products of *ADRB2* for the 27^th^ amino acid digested with *ItaI*. Upper panel: for hominoids. Lane 1: PCR products of a human; not digested (353 bp). Lane 2: fragments of human *Gln27/Glu27* (229, 174, 97, 55 and 27 bp which was undetectable at this concentration). Lane 3: fragments of human *Gln27/Gln27* (174, 97, 55 and 27 bp). Lane 4 to lane 7: fragments from *P. troglodytes*, *G. gorilla*, *P. pygmaeus*, and *H. agilis*, respectively (229 bp instead of 174 and 55 bp). Lower panel: for macaques. Lane 1: PCR products of *M. mulatta*; not digested (222 bp). From lane 2 to lane 6: fragments from *M. mulatta*, *M. fuscata*, *M. fascicularis*, *M. nemestrina*, and *M. radiata*, respectively (168 bp instead of 113 and 55 bp).

The upper panel of Figure 3A shows the predicted restriction map of *ADRB2* digested by *ItaI* (GCAGC) for hominoids from the DNA sequences. The upper panel of Figure 3B shows the obtained RFLP pattern of *Ita*I-digested PCR product of *ADRB2* containing the *Gln27Glu* site of hominoids. All apes, *P. troglodytes*, *G. gorilla*, *P. pygmaeus*, *and H. agilis* were homozygous for *Glu27* because a 229 bp fragment was obtained instead of 174 and 55 bp fragments (lanes 4–7). This region of the macaques could not be amplified with the primer set for hominoids but could be amplified with the new primer set to produce a 222 bp amplicon (Fig. 1 from primer 27MF to 27MRc). The predicted restriction map digested by *ItaI* for macaques is shown in the lower panel of Figure 3A. The *Ita*I-digested macaque amplicon yielded fragments of 168 bp for the homozygous *Glu* allele instead of 55 and 113 bp fragments (lower panel of Fig. 3B). All hominoids and macaques were homozygous for *Glu27* in *ADRB2* (Table 1).

**Figure 4:**
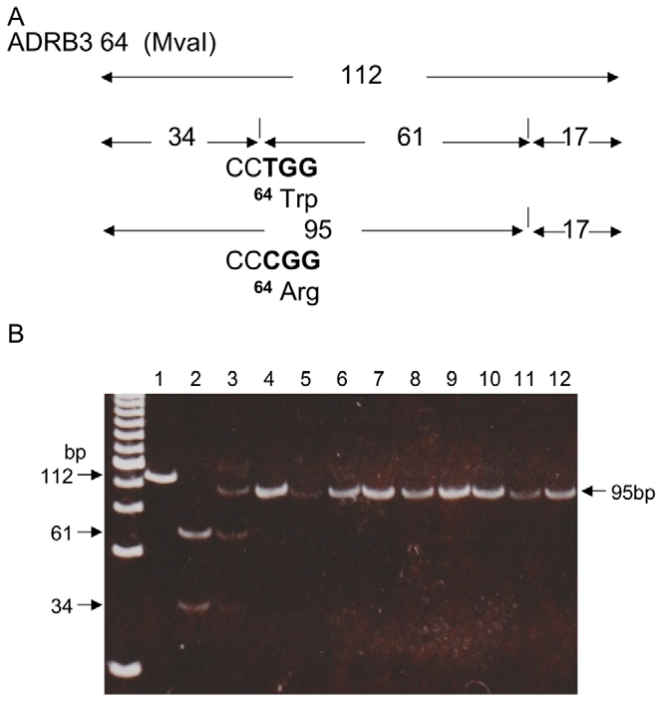
All NHP had *Arg64* allele in *ADRB3*. A) Restriction map of *ADRB3* digested with *Mva*I for humans and NHP. B) RFLP patterns of PCR product of *ADRB3* digested with *Mva*I for humans and NHP. Lane 1: PCR products of a human; not digested (112 bp). Lane 2: fragments of the human *Trp64/Trp64* (61, 34 and 17 bp which was undetectable at this concentration.) Lane3: fragments of human *Arg64/Trp64* (95, 61, 34 and 17 bp). Lane 4 to lane 12: fragments from *P. troglodytes*, *G. gorilla*, *P. pygmaeus*, *H. agilis*, *M. mulatta*, *M. fuscata*, *M. fascicularis*, *M. nemestrina*, and *M. radiata*, respectively (95 bp instead of 61 and 34 bp).

The nucleotide sequences of 70 bp including codon 64 of *ADRB3* in *G. gorilla* (AB669105), *P. pygmaeus* (AB669106), *H. agilis* (AB669107), *M. fascicularis* (AB669108), *M. fuscata* (AB669109), *M. nemestrina* (AB669110), and *M. radiata* (AB669111) were determined. All of them confirmed that the restriction site with *MvaI* was CCCGG not CCTGG together with *P. troglodytes* (ENSPTRG00000024086) and *M. mulatta* (ENSMMUG00000005876). The restriction map digested by *Mva*I (CC (T/A) GG) of this region was predicted from these sequences among humans and NHP, as shown in Figure 4A. Since all digested *ADRB3* amplicons from NHP gave a 95 bp fragment but no 61 and 34 bp fragments, as shown in lanes 4 to 12 in Figure 4B, they had a homozygous form of *Arg64*, as shown in Table 1.

**Figure 5:**
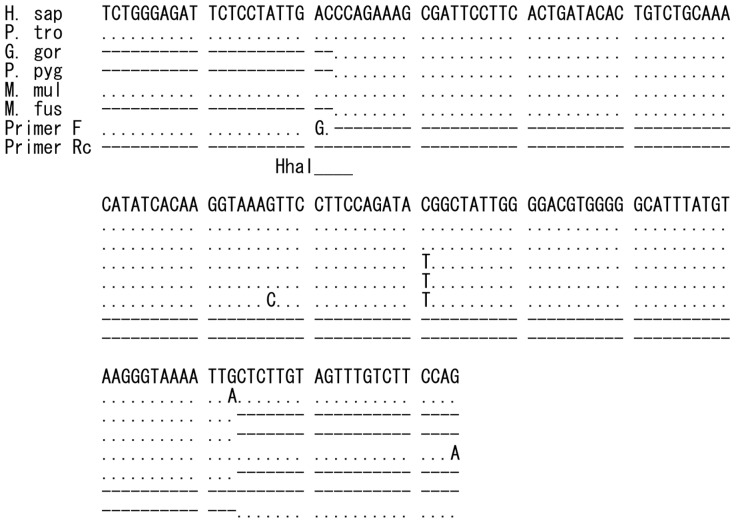
Nucleotide sequences in *PPARG* of primates. The determined sequences of *G. gorilla* (AB669114), *P. pygmaeus* (AB669115), and *M. fuscata* (AB669116) together with the Ensembl database of human (ENSG00000132170), *P. troglodytes* (ENSPTRG00000014632) and *M. mulatta* (ENSMMUG00000007191). Underline shows the restriction site (GCGC) with *HhaI*. The restriction site region of *M. fascicularis* (AY048695) from the GenBank database was the same as in other macaques. One nucleotide of primer F was changed to create the restriction site of *HhaI* [34].

Figure 5 shows the nucleotide sequences of *PPARG* between primers. RFLP method by digestion with *HhaI* (GCGC) was carried out according to Hara et al. [34]. All examined NHPs had 154 bp instead of 23 and 131 bp fragments, indicating the thrifty type, *Pro12* allele (Table 1).

Consequently, previously unknown features were identified that all NHPs had thrifty-type alleles, *Gly16* and *Glu27* in *ADRB2*, *Arg64* in *ADRB3*, and *Pro12* in *PPARG*.

## Discussion

The adrenergic receptors, *ADRB2* and *ADRB3*, have an important role in lipolysis and thermogenesis, and the polymorphism of these genes causes differences in energy expenditure. Alleles of both *Glu27* in *ADBR2* and *Arg64* in *ADRB3* are associated with obesity and/or non-insulin-dependent diabetes mellitus (NIDDM) in humans.

Neel [35] proposed the concept of a thrifty gene in humans, which nowadays causes diabetes mellitus given the high availability of food, but was beneficial in the periods of feast or famine experienced in hunter-gatherer cultures. Our present study shows that all examined NHP including apes had the thrifty type in the functional hot spots, *Glu27* in *ADRB2* and *Arg64* in *ADRB3*. This means that these thrifty-type alleles did not develop at the time of hunter-gathering in humans, but rather that NHP had them already.

Although it is not clear whether the *Gly16* in *ADRB2* is related to obesity, polymorphism of this site was not found in the non-human hominoids; all of them had *Gly16*. Since the restriction site of *BsrD*I was not retained in the macaques, we did not analyze them. However, all macaque species for which the nucleotide sequence was determined had the *Gly16* allele (see Fig. 1).

**Figure 6:**
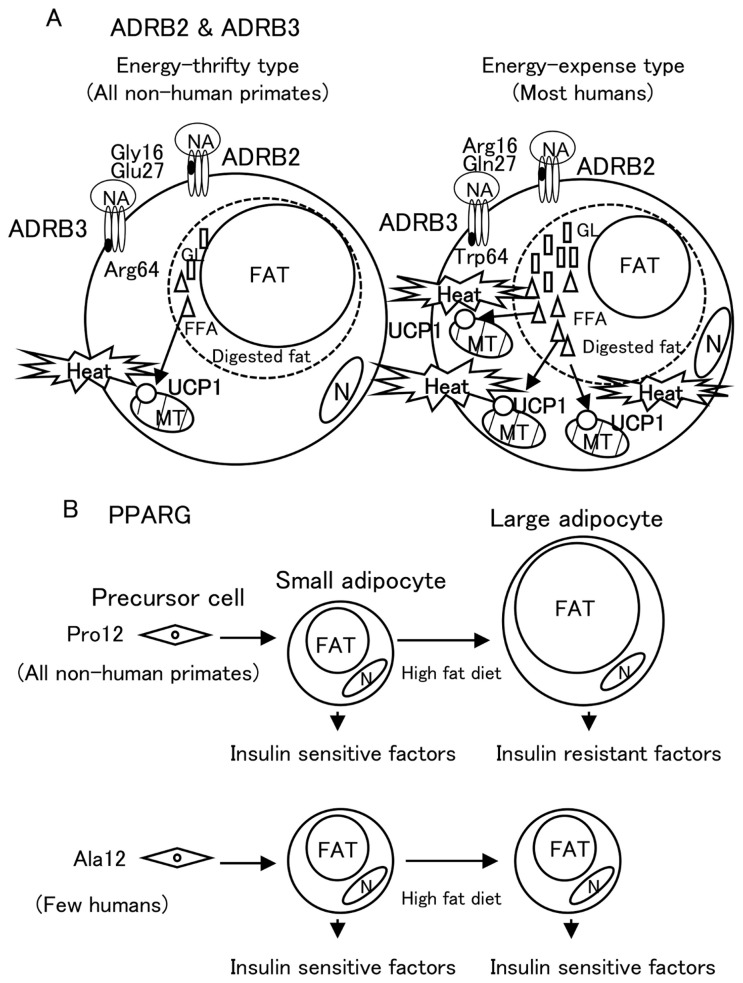
Summaries of effects of SNPs in *ADRBs* and *PPARG*. A) ADRBs with energy expense-type allele found only in humans stimulate the digestion of accumulated fat in adipocytes at a higher level. A lot of generated free fatty acids (FFA) stimulate both transcription of the uncoupling protein (*UCP1*) gene and the activity of UCP1 proteins, which generate heat in mitochondria. GL: glycerol. B) PPARG activates differentiation from precursor cells into small-sized adipocytes, which secrete insulin-sensitive factors. PPARG with *Ala12* found only in humans causes reduction of transcriptional activity of PPARG and leads to protection from high-fat-diet-induced hypertrophy of adipocytes, which secrete insulin-resistant factors [30], [34].

PPARG activates differentiation of precursor cells of adipocytes to the small-sized adipocytes, which secrete factors that prevent diabetes mellitus. If rodents that possess *PPARG* with *Pro12* consume a high-fat diet, the small-sized adipocytes accumulate more fat to change to hypertrophic adipocytes, which secrete factors that promote diabetes mellitus, such as TNFα, resistin and free fatty acids. However, heterozygous *PPARG*-deficient mice showed protection from insulin resistance induced by a high-fat diet and an increased number of small-sized adipocytes [31]. The number of small-sized adipocytes is thought to increase with a high-fat diet in the case of *PPARG* with *Ala 12* allele, the same as in heterozygous *PPARG* deficiency, because the transcriptional activity of PPARG with *Ala12* was shown to be lower [33]. Actually, human subjects with *Ala12* showed insulin sensitivity and the frequency of *Ala12* allele was lower in diabetics than in healthy humans [34]. Thus, *PPARG* with *Pro12* allele is thought to be a thrifty type. All NHP were found to have the thrifty type *Pro12* allele in *PPARG*. The differences of these functions between alleles and results are summarized in Figure 6. These thrifty-type alleles, Glu27 in ADRB2, Arg64 in ADRB3 and Pro12 in PPARG, are preserved in some present-day humans and cause NIDDM in food-abundant conditions, as presumed by Neel. Antagonism of insulin, as mentioned by Neel, might suggest insulin-resistant substances secreted from hypertrophic adipocytes, such as TNFα, resistin and free fatty acids.

### Why do non-human primates retain thrifty genes?

All the examined NHP showed the thrifty type at these four hot spots. However, it might be necessary to confirm the current findings using much larger sample sizes as, in this study, the sample sizes, especially those for the great apes, were limited, because these DNA samples were nonrelated.

The food abundance and/or food intake of NHP changes seasonally not only in temperate regions [36] but also in tropical regions [37]–[43]. Accordingly, body weight [44], fat deposition [45], and energy-consuming activities, such as traveling [46], [47], are reduced during periods when food is scarce. Therefore, if an individual with the energy-loss types, *Gln27* in *ADRB2* or *Trp64* in *ADRB3*, happened to appear, they might be at a fitness disadvantage because they would lose energy by higher lypolysis and thermogenesis and could not accumulate enough fat in the adipose tissues to survive during food shortages. *Pro12* allele in PPARG might help in the accumulation of more fat to produce large adipocytes during the higher-fat-diet season, which could be utilized in subsequent seasons with a food shortage.

### Why did humans evolve energy-loss genes?

As the *Gln27* allele of *ADRB2* and the *Trp64* allele of *ADRB3* are found all over the world in human populations, these alleles might have appeared before the migration out of Africa and now be present in many populations. Cagliani et al. [27], [28] and Wilson et al. [28] speculated that the divergence occurred around 1.9 MYA and that, from Fisher's exact test of *ADRB2*, this gene may be associated with balancing selection or relaxed constraint. The lipolysis activity of ADRB3 with the *Trp64* allele is higher than that with *Arg64*. The production of second messenger, cAMP [8], [48], and glycerol by lipolysis was 1.5-fold higher in those homozygous for *Trp64* than in those homozygous for *Arg64* [9]. Blood glycerol level and hydroxybutyrate level were higher, that is, higher lipolysis, in humans with *Gln27* in ADRB2 [49]. Free fatty acids produced by lipolysis by stimulation of ADRBs accelerate transcription of the *UCP1* gene and increase the thermogenesis activity of the UCP1 protein.

The human lineage is thought to have diverged from the chimpanzee lineage 6 MYA. It took a long time for the ancestors of humans to arrive at the savanna from the tropical forests and to live as hunter-gatherers. Fossils of *Ardipithecus* dated to 5.6 MYA [50] and 4.4 MYA [51], [52] indicated that they combined arboreal palmigrade clambering with terrestrial primitive bipedality. Although the footprints of bipedals dated 3.6 MYA were found at Laetoli, Tanzania [53], *Australopithecus* from 3.2 MYA might have exhibited a mixture of terrestrial and arboreal locomotion [54]. The oldest stone tools have been dated to between 2.6 and 2.5 MYA [55]. *Homo habilis*, which emerged around 2.3 MYA [56], had a bigger brain (600–800 ml) [57] and could make unifacial core stone tools [58], but they were thought to be able to eat meat only by scavenging or hunting small animals [59] because their bipedal locomotion was still incomplete [60]–[62].

*Homo erectus*, which emerged 1.8 MYA, was the first *Homo* that could walk completely bipedally [63]–[65]. They could hunt large animals in the savanna after chasing them over long distances since their brain size had increased dramatically compared with that of *habilis*, from 800 to 1200 ml [57]. They could also make hand axes (stone tools in Acheulean culture) [66] and might have had sufficiently high intelligence to enable advanced cooperation. They migrated out of Africa to Asia and Europe 1.8 -1.0 MYA [67], [68], but they were not the origin of modern humans.

Mitochondrial DNA analysis of the present-day world population has suggested that a small proportion of the descendants of *Homo erectus* migrated from Africa 0.15 MYA and spread throughout the world 60–70,000 years ago [69]. This theory about the single origin of modern humans is supported by nuclear DNA analysis [70], [71].

The human ancestors who left tropical rainforests for savanna and developed new features in their heat-regulation systems owing to stronger solar radiation, such as reduction of body hair and increased evaporation of water through an increased number of eccrine glands, might have been *Homo erectus* who were fully bipedal and had sufficiently big brains for making tools and cooperating at an advanced level. Apocrine glands are located deep in the dermis and their ducts lead to hair follicles associated with sebaceous glands. Eccrine glands, which lie in a shallow portion of the dermis between hair follicles and have ducts that open to the surface of the skin directly, are not associated with hair follicles, and can secrete water including sodium chloride. The proportion of eccrine glands to apocrine glands was found to be around 100% in humans but 70% in *G. gorilla* and *P. troglodytes* [72]. Baboons and *P. troglodytes* increase the activity of sweat glands and the respiratory rate upon an increase of environmental temperature to 40°C. Humans, however, do not increase respiratory frequency but instead increase the activity of sweat glands [73], [74]. The hair density of NHPs decreases systematically with increasing body surface area, that is, massive primates have fewer hairs to lose metabolically generated heat owing to a relatively low ratio of surface/mass; however, *Homo erectus*, unique among primates, had hairless skin except in small areas because the evaporation of water from the developed eccrine glands might have occurred efficiently owing to a loss of hair for cooling during overheating among those who lived under the high radiant heat loads of the savanna [75], [76].

It is known that a glacial period started around 2.4 MYA, which was identified by carbonate content and oxygen isotope analysis of cores from the North Atlantic [77]. The global temperature changed periodically. A deep (more than 150 m) and wide (several 100 m^2^) freshwater lake appeared twice in the South Kenyan and Tanzanian Rift Area (1.9-1.7 MYA and 1.0 -0.7 MYA) owing to the precessional forcing and progressive rifting of East Africa [78]. Extreme aridity occurred and C4 plants grew in the absence of these wet phases [79]–[81]. Almost the same changes of oxygen isotope as occurred during the last glacial period also occurred at the beginning of the glacial periods 2.4-2.3 MYA and 2.1-2.0 MYA; slightly smaller changes also continued periodically until the last glacial period [77]. The lowest global average temperature in the glacial period was speculated to be as much as 8°C lower than the present value, as determined from the relationship between temperature and oxygen isotope, 1.4°K/‰ [82], in the ice cores from several sites (Greenland [83], Antarctica [84], and Mt. Kilimanjaro [85]) and North Atlantic deep sea drilled cores [77]. As the highest and lowest temperatures at Arusha, 150 km from Olduvai Gorge in Tanzania, are 23°C and 13°C in July, the winter and dry season in the present day, *Homo erectus* might have experienced temperatures of around 5°C at night.

Consequently, *Homo erectus* might have felt cold with large temperature differences at night, especially in the glacial period. It might have been hard to maintain their body temperature at night, especially for children. Present-day children, however, have four times more brown adipose tissue than adults until the age of ten years old, even though adults keep half of their brown adipose tissue in the abdominal area [86]. The brown adipose tissue expresses UCP1 and might be able to produce heat. Fortunately, there was a mutation of ADRB2 from a thrifty type to an energy expense type 1.9 MYA [27], [28]. Noradrenaline from the sympathetic nervous system is secreted by cold stimulation and produces much more heat through the *Gln27* allele of ADRB2 and the *Trp64* allele of ADRB3, which helps maintenance of body temperature of *Homo erectus* more than the *Glu27* allele or the *Arg64* allele.

However, the increase of thermogenesis might have caused increased total energy expense, which might have required increased energy production. *Homo erectus* could hunt large animals with high nutritional value using hand axes and narrow slivers of rock, as well as by utilizing full bipedal locomotion. Wrangham [87] proposed that *Homo erectus* might have cooked using fire on the basis of several findings: large anatomical differences from *Homo habilis*, especially smaller tooth size, less flared rib cage, and narrower pelvis indicating a smaller gut [64], [88], which could digest cooked food easier than raw food and absorb more energy. If this is true, we can state that *Homo erectus* could survive by hunting animals using tools and by using fire to obtain more energy in spite of the single nucleotide changes in *ADRB2* and *ADRB3* genes that conveyed a greater energy loss but high thermogenesis to protect entrails from cold in the glacial period. In addition, fire might have warmed their bodies during the night.

During the period when *Homo erectus* remained in Africa from 1.8 to 0.15 MYA, these energy expense-type alleles spread among them and, when they migrated out of Africa, every subsequently formed population might have had both thrifty-type and expense-type alleles.

In conclusion, the energy expense-type alleles, *Gln27* of *ADRB*2, *Trp64* of *ADRB3*, and *Ala12* of PPARG, which induce small-sized adipocytes, are specific SNP that have only accumulated in humans, and convey abilities of both high energy loss and high thermogenesis, and less accumulation of fat in adipocytes. The advantage of energy expense-type alleles of ADRB2 and ADRB3 for increasing body temperature of *Homo erectus* and the ancestors of modern humans in the glacial period may explain the higher frequencies of energy expense-type alleles than thrifty-type alleles.

## Materials and Methods

This study was carried out within the ethical guidelines and framework of Kyoto University and was approved by the Primate Research Institute, Kyoto University, and Kumamoto Sanctuary. Macaques in the Primate Research Institute, Kyoto University, were bred following the third edition of The Guide for the Care and Use of Laboratory Primates (Primate Research Institute, Kyoto University) in accordance with the National Research Council Guidelines “Guide for the care and use of laboratory animals 1996”. Individual cages for macaques with a body weight (BW) of 3–10 Kg measured 0.39 m^2^ floor area ×76.2 cm height, and those for macaque with BW of 10–15 Kg were 0.54 m^2^×81.3 cm. The temperature was set at 27°C in summer and 23°C in winter for the long-tailed macaques, and for other macaques at 20°C in winter. Humidity was set at 40–70%.

Macaques in an open-air corral were bred at less than 30 heads per 500 m^2^. They were provided with a jungle gym made of wooden logs with two small rooms to avoid rain or low temperature, a stream and a pond with small fish. Feeding was as follows: Japanese macaques received 48–55 kcal/kg/day monkey chow and rhesus macaques received 40–50 kcal/kg/day, with feeding twice a day and some sweet potatoes every two days. Wheat or dried soybeans were fed to macaques in the open-air corral twice a week. Some blood samples of the macaques bred at the PRI, Kyoto University, were taken before 1996 when all procedures were conducted according to the second edition of the Guide for Care and Use of Laboratory Primates following the guidelines issued by the NIH in 1985. The cages were wider and higher than those prescribed by the third edition from 1996.

All procedures for chimpanzees were conducted according to the third edition of the Guide for the Care and Use of Laboratory Primates (Primate Research Institute, Kyoto University) and the Guidelines for Care of Chimpanzees (Kumamoto Sanctuary). All chimpanzees lived in social groups. They could spend time in an outdoor playground where a jungle gym with fire hoses and ropes to climb had been set up. Trees and grass had also been planted. The ground area was 15–60 m^2^ per head. Their basic food per day was monkey chow (PS by Oriental Yeast Co., Ltd., and Monkey Bit by Nosan Corporation) at 300–500 g depending on the BW, one banana, two oranges (around 500 g), sweet potatoes at 500 g, carrots at 150 g, cabbage at 500 g, and seasonal vegetables or fruit at 500 g. Wild grass and twigs with leaves were given every day. Soybeans, sunflower seeds, peanuts with shells, honey, fruit juice and sugar cane were set in puzzle feeders to be eaten freely. Chimpanzees were fed more than five times per day.

Blood samples of non-human primates were obtained from 30 chimpanzees (one *Pan troglodytes schweinfurthii*, 29 *Pan troglodytes verus*) from The Kumamoto Sanctuary, Kyoto University (previously named The Chimpanzee Sanctuary Uto) (permission numbers P1988-08 [89]). Blood samples of sixteen orangutans (*Pongo pygmaeus*) were collected for a biochemical health check, testing for hepatitis virus and evolutionary study with permission from the Sepilok Rehabilitation Center in West Malaysia in 1988 [90]. Three agile gibbons (*Hylobates agilis*) were from Ragunan Zoo and 12 pet monkeys were from a field survey at Pangkalan Bun in Kalimantan, Indonesia, with the permission of Bogor Agricultural University and supported by Competitive Research Grant from the Ministry of Education and Culture of Indonesia awarded to Dr. Bambang Suryobroto (No. 03/P2IPT/DPPM/96/PHB I/5/1996) and by Grant-in-Aid for Scientific Research (Overseas Scientific Survey) No. 08041147 from the Ministry of Education, Culture, Sports, Science and Technology of Japan in 1996 [91]. 108 macaques including rhesus macaques (*Macaca mulatta*) that originated in China and India, Japanese macaques (*Macaca fuscata fuscata*) that originated in Wakasa, Arashiyama, Awajishima, and Koshima, Japan, long-tailed macaques (*Macaca fascicularis*) that originated in Indonesia, bonnet macaques (Macaca radiata) from India and pig-tailed macaques (*Macaca nemestrina*) were from the Primate Research Institute, Kyoto University.

Muscle samples of 8 Western lowland gorillas (*Gorilla gorilla gorilla*) were imported (Import permission No. JP9129795, June 25, 1992) from the Gorilla Orphanage in Brazzaville, the Republic of Congo, for clinical pathological study and evolutionary study of great apes in Africa (Grant-in-Aid for Scientific Research from the Ministry of Education, Culture, Sports, Science and Technology (No. 04044153)) in 1992 by the late Professor S. Hayama and his team. Many baby gorillas had died and been kept in a stock freezer in the Gorilla Orphanage in Brazzaville. Prof. Hayama and his team went to the Republic of Congo in 1991 and 1992 at the request of the Government of the Republic of Congo and the Gorilla Rescue Center (Dr. Mark Attwater) to investigate the causes of death. They carried out anatomical study in Brazzaville and brought back several organs including muscles for more precise clinical pathological studies and evolutionary studies in Japan. It was found that the gorillas had suffered from helminthiasis, dehydration, anorexia nervosa, influenza viral pneumonia and acute anterior poliomyelitis [92], [93].

The blood samples were not originally collected for the present study, but as part of routine health examinations and field survey by the late Professor Osamu Takenaka. During these examinations, chimpanzees were sedated with oral midazolam (1 mg/kg) or droperidol (0.2 mg/kg), and their blood was collected while they were anesthetized with ketamine hydrochloride (7 mg/kg) or a combination of ketamine hydrochloride (3.5 mg/kg) and medetomidine hydrochloride (0.035 mg/kg). The blood samples of other primates were collected under general anesthesia with ketamine hydrochloride (5–10 mg/Kg BW)+atropine (0.02–0.05 mg/Kg BW) by intramuscular injection.

PCR was performed using 10 ng of DNA and Ampli Taq^R^ Gold DNA polymerase (Applied Biosystems Co. Ltd.) in a total volume of 25 µl under the following conditions: *ADRB2* fragment with the *Arg16Gly* variant site was amplified in accordance with Large et al. [20] with modification of the forward primer to create the restriction site for *BsrD*I (GCAATGNN). *ADRB2* amplicon containing *Gln27Glu* substitution site was amplified by the method of Large et al. [20] using a primer set of 5′-GAATGAGGCTTCAGGCGTC-3′ (forward) and 5′-GGCCCATGACC AGATCAGCA-3′ (reverse) in the presence of dimethylsulfoxide for hominoids, and other primers of 5′-CAGACAGTGCGCTCACCTGCCAGA-3′ (forward) and 5′-ACGCTCGAACTTGGCAATGGCT-3′ (reverse) for macaques because of a lack of amplification with the primers for hominoids. *ADRB3* PCR product containing the *Trp64Arg* polymorphism site was amplified at 95°C for 9 min, then 32 cycles of 95°C for 1 min, 55°C for 1 min, and 72°C for 2 min, followed by 72°C at 5 min using primers of 5′-TGGGAGGCAACCTGCTGGTCAT-3′ (forward) and 5′-AGGAGTCCCATCACCAGGTC-3′ (reverse). PCR product of *PPARG* containing *Pro12Ala* polymorphism site was amplified by the method of Hara et al. [34] using the same forward primer, which creates the restriction site of *HhaI*. However, the reverse primer region includes one more T in the sequence of *H. sapiens*, *P. troglodytes*, *and M. mulatta* from the Ensembl database, so the reverse primer including one more complementary nucleotide A was used (Fig. 5).

*ADRB2* fragment with the *Arg16Gly* variant site with 5 U of *BsrD*I (New England Bio Labs Co. Ltd.) at 65°C for 1.5 hrs. *ADRB2* amplicon for the *Gln27Glu* substitution site (5 µl) was digested with 5 U of *Ita*I (Roche Applied Science Co. Ltd.) at 37°C for 1 hr. *ADRB3* PCR product containing the *Trp64Arg* polymorphism site (5 µl) was digested with 5 U of *Mva*I (CC(A/T)GG) (TAKARA Co. Ltd.) at 37°C for 1 hr and *PPARG* amplicon containing *Pro12 Ala* variant site was digested with 5 U of *HhaI* (GCGC) (TAKARA Co. Ltd.) at 37°C for 3 hrs. The digested fragments were separated by 12% polyacrylamide gel electrophoresis and visualized using SYBR Green I (Cambrex Co. Ltd.) under a UV illuminator.

DNA sequence analysis was performed to confirm the restriction site of PCR products of *ADRB2*, *ADRB3*, and *PPARG* using an ABI PRISM™ 310-20 Genetic Analyzer.
